# Correction to: Effects of the COVID-19 pandemic on trauma-related emergency medical service calls: a retrospective cohort study

**DOI:** 10.1186/s12873-021-00511-6

**Published:** 2021-11-19

**Authors:** Michael Azbel, Mikko Heinänen, Mitja Lääperi, Markku Kuisma

**Affiliations:** 1 Prehospital Emergency Care Services, Lapland Central Hospital, P.O. Box, 8041, FI-96101 Rovaniemi, Finland; 2 Department of Emergency Medicine and Services, Helsinki University and Helsinki University Hospital, P.O. Box 340, FI-00029 Helsinki, Finland; 3 Trauma Unit and Helsinki Trauma Registry, Helsinki University Hospital, P.O. Box 266, FI-00029 Helsinki, Finland; 4 Department of Orthopaedics and Traumatology, University of Helsinki and Helsinki University Hospital, Helsinki, Finland

**Correction to: BMC Emergency Medicine 21, 102 (2021)**

**https://doi.org/10.1186/s12873-021-00495-3**

The original article [1] mistakenly removed negative values in the ‘Change % (IQR) column of Tables 1-3.

These values have now been corrected.
